# Stroke Riskometer Application (SRA™) influence on lifestyle changes of home bound familial Malaysian stroke caregivers: a randomised controlled trial in a primary care based longer term stroke care facility

**DOI:** 10.1186/s12875-023-02138-x

**Published:** 2023-09-08

**Authors:** Radhiyah Hussin, Aznida Firzah Abdul Aziz, Mohd Fairuz Ali, Ezura Madiana Md Monoto, HS Arvinder-Singh, Alabed Ali Ahmed Alabed, Wan Asyraf Wan Zaidi, Norlinah Mohamed Ibrahim, FRCPE

**Affiliations:** 1 Department of Family Medicine, Faculty of Medicine, Universiti Kebangsaan Malaysia, Level 14, Preclinical Block, Jalan Yaacob Latif, Bandar Tun Razak, 56000 Kuala Lumpur, Cheras Malaysia; 2 Klinik Kesihatan Tanglin, Ministry of Health, Jalan Cenderasari, Kuala Lumpur, Malaysia; 3 Community Health Department, Faculty of Medicine, Universiti Kebangsaan Malaysia, Kuala Lumpur, Cheras Malaysia; 4 Institute of Clinical Research, Sungai Buloh Hospital, Sungai Buloh, Selangor, Malaysia; 5 Community Medicine Department, Faculty of Medicine, University of Cyberjaya, Cyberjaya, Selangor Malaysia; 6 Neurology Unit, Department of Internal Medicine, Faculty of Medicine, Universiti Kebangsaan Malaysia, Kuala Lumpur, Cheras Malaysia

**Keywords:** Stroke, Caregiver, Informal caregiver, Stroke rehabilitation

## Abstract

**Background:**

In countries where access to Specialist stroke care services are limited, primary care physicians often manage stroke patients and the caregiving family members. This study aimed to evaluate the impact of Stroke Riskometer Application (SRA™) on promoting healthier lifestyles among familial stroke caregivers for primary prevention.

**Methods:**

A parallel, open-label, 2-arm prospective, pilot randomised controlled trial was conducted at a long-term stroke service at a university based primary care clinic. All stroke caregivers aged ≥ 18 years, proficient in English or Malay and smartphone operation were invited. From 147 eligible caregivers, 76 participants were randomised to either SRA**™** intervention or conventional care group (CCG) after receiving standard health counselling. The intervention group had additional SRA™ installed on their smartphones, which enabled self-monitoring of modifiable and non-modifiable stroke risk factors. The Stroke Riskometer app (SRA^TM^) and Life's Simple 7 (LS7) questionnaires assessed stroke risk and lifestyle practices. Changes in clinical profile, lifestyle practices and calculated stroke risk were analysed at baseline and 3 months.

The trial was registered in the Australia-New Zealand Clinical Trial Registry, ACTRN12618002050235.

**Results:**

The demographic and clinical characteristics of the intervention and control group study participants were comparable. Better improvement in LS7 scores were noted in the SRA™ arm compared to CCG at 3 months: Median difference (95% CI) = 0.88 (1.68–0.08), *p* = 0.03. However, both groups did not show significant changes in median stroke risk and relative risk scores at 5-, 10-years (Stroke risk 5-years: Median difference (95% CI) = 0.53 (0.15–1.21), *p* = 0.13, 10-years: Median difference (95% CI) = 0.81 (0.53–2.15), *p* = 0.23; Relative risk 5-years: Median difference (95% CI) = 0.84 (0.29–1.97), *p* = 0.14, Relative risk 10-years: Median difference (95% CI) = 0.58 (0.36–1.52), *p* = 0.23).

**Conclusion:**

SRA™ is a useful tool for familial stroke caregivers to make lifestyle changes, although it did not reduce personal or relative stroke risk after 3 months usage.

**Trial registration:**

No: ACTRN12618002050235 (Registration Date: 21^st^ December 2018).

## Background

The burden of caring for long term stroke survivors in Malaysia, like in most other Asian countries, falls mainly on the family and extended family members [1, 2]. The high strain of caring for family members with stroke makes the caregiver population susceptible to stroke due to stress and possibly suffering from non-communicable disease (NCD) themselves [1, 2]. Due to the increasing prevalence of NCDs in the world, primary prevention is the most effective measure to reduce the burden of NCDs [3]. As stroke shares many risk factors with other major NCDs, such as ischaemic heart disease, vascular dementia, diabetes and some type of cancer, measures to control stroke risk factors and improve primary stroke prevention are likely to reduce burden from other NCDs [4, 5].

Globally, mobile phones have achieved wide internet coverage at an unprecedented rate, with 94.6% of smartphone users in 2018 accessing internet via mobile phones [6]. Hence, applications or apps have become increasingly prevalent among users. Malaysia’s expansion of its smartphone market is expected to hold steady at around 1 percent annually in the coming years. There has been a surge of health-related mobile phone apps in recent years. Apps used in health care settings have several functions or address different aspects, such as information and time management, communication and consulting, patient management and monitoring [7, 8]. According to Jing Zhao et al. 17 studies reported statistically significant effects in the direction of targeted behaviour change, using apps, with the commonest being self-monitoring. One of the primary benefits of apps is their potential for incredibly high population coverage [9]. With mobile phone use reaching near saturation among some populations, particularly young adults, and the high rates of consumer acceptability, apps that offer even a small health benefit could still be a valuable public health intervention tool or medium, if the population-level reach is high enough [10, 11]. Reliable and medically accurate health-related apps are rendered more reachable and convenient [12]. Concurrently, the Internet and mobile interventions improved important lifestyle behaviours up to six months post-stroke [13].

The Stroke Riskometer application (SRA™) is a novel, free, evidence based, validated and internationally endorsed and widely translated (19 languages, including Malaysian) tool to address the gaps in stroke knowledge and primary stroke prevention [10]. The app estimates the risk of stroke and provides individual risk factor-tailored information on healthy lifestyle and other risk factors management to reduce risk from NCDs including stroke. It has a goal setting and push notification options [10]. All users’ data can be saved on the users’ smartphones for comparison over time and self-monitoring progress in the management of risk factors. The app has been translated and validated for use in Malaysia.

Stroke patients in Malaysia are mainly home-bound and are cared by family members [1, 11]. Familial risk of stroke risk factors among caregivers may be missed, and only recognised when complications manifest, rendering caregivers incapacitated. Despite the high numbers of informal caregivers who suffer economic, physical and emotional burden, data on supporting usage of app and its cost effectiveness evaluation is scarce [14]. In 2011, WHO issued a statement supporting the use of mobile and wireless technologies to achieve health objectives (mHealth) and transform the face of health service delivery across the globe [8]. It was found that the most effective apps were those that used goal setting and self-monitoring approach [10, 13]. A pilot randomised controlled trial conducted among first time users of the same SRA™ showed improvement of mean cardiovascular health after exposure to the app [15]. Hence, this study aims to investigate effects of the SRA™ on informal stroke caregivers’ risk of stroke and risk factors management, i.e. to assess the impact of primary prevention delivery via a smartphone application to influence familial stroke caregivers toward healthier lifestyle practices.

## Methods

We conducted a pilot 2-arm parallel, open-labelled randomized controlled trial (RCT). Informal caregivers of stroke patients who attended a pioneer primary care-based longer-term stroke care service at Klinik Primer PPUKM Cheras (aka Klinik Lanjutan Strok, KLS) during December 2018 to May 2019 period were approached for this study. The KLS was formed to provide longer-term stroke care monitoring for post discharge stroke patients, in a shared care initiative between primary care and Stroke Specialist team [16]. This clinic remains the only primary care clinic to provide an integrated post stroke care service which actively involves multidisciplinary team contribution for patients in the community.

### Sample size calculation

As this study was a pilot study with numerical outcomes, based on Billingham et al.’s [17] recommendation, a minimum of 30 participants were required for each group. This decision was also made based on practical factors such as willingness of the caregivers to participate in the study, attendance rate of patients, recruitment and monitoring to be done at same follow up visit for the stroke patient.

### Randomisation and masking

Computer generated number was used for simple randomisation to identify potential participants from the KLS appointment list, and those eligible were contacted via telephone by researcher. Familial or blood-related caregivers of stroke patients aged ≥ 18 years old, owns a smartphone and literate in either Malay or English were recruited. Pregnant caregivers or caregivers with possible depression (i.e. respondents screened positive using the Two Questions With Help Questionnaire, TQWHQ) were excluded. Shortlisted participants were contacted by the researcher and briefed about the study. Participants who agreed to participate signed the informed consent document.

Participants were asked to provide details of their sociodemographic data (proforma), stroke risk (SRA™, 20 questions) and lifestyle practices (LS7 questionnaires), and were then interviewed by the researcher to verify their risk factors for stroke (i.e. hypertension, diabetes, dyslipidaemia, previous history of stroke), including physical examination (i.e. blood pressure, body mass index) and reviewing blood tests for total cholesterol and fasting blood sugar levels within the last 6 months. The researcher then used the SRA™ to calculate stroke risk and informed study participants about the findings. All participants in the usual care and SRA™ intervention group received standard counselling and explanation about their stroke risk. Once briefed about their stroke risk, they were randomly assigned to either intervention (SRA™) or conventional care group (i.e., education pamphlet from Ministry of Health, MOH).

An assistant researcher, who was not part of the study, managed allocation concealment. This involved enrolling participants and assigning them to different treatments using a randomly generated code from sealed envelopes. The main researcher created the code before the study and kept it concealed until participants were recruited after baseline measurements.

Due to the nature of the intervention, masking was not possible. Caregivers in the intervention group received the SRA™ which was downloaded onto their smartphones by the researcher during intervention assignment, in addition to the standard health education (pamphlet). The pamphlet, which is authored by the MOH contains general healthy lifestyle advice and did not contain any information or endorsement on usage of app to reduce risk of stroke or NCD (MOH 2012).

To prevent contamination of data among participants during the study, caregivers were asked if they were aware or have downloaded any healthcare application on stroke risk reduction prior to the study and during exit visit assessment. Study participants were then assessed three months later (exit visit assessment), during a face-to-face evaluation visit or via telephone by the researcher based on participants’ availability for assessment.

### Study tools and questionnaires

#### Stroke Riskometer Application (SRA™)

This smartphone application was developed by AUT University of Auckland, New Zealand and is fully validated [15]. Permission to use the app was obtained. This app contains 20 questions specifically designed to assess the users’ / participants’ modifiable and non-modifiable risk factors [15]. The user is asked to share their personal health information. The collected data consisted of three domains: Part A: Non modifiable risk factors (i.e., age, gender and race); Part B: Modifiable risk factors: Blood pressure, Weight and height, Smoking status, excessive alcohol consumption, physical activities, healthy diet, family history of stroke, diabetes, hypertension, atrial fibrillation, dementia or risk of dementia; Part C: Percentage of stroke risk and relative risk of getting stroke compared to someone of the same age, gender and ethnicity was calculated in 5 and 10 years.

#### Life’s Simple 7 (LS7)

This a metric based scale used by the American Heart Association (AHA) to guide people to achieve ideal cardiovascular health by making lifestyle changes on seven identified risk factors [18]. Permission to use this scale was obtained from the AHA. The components include: smoking, diet, physical activity, body mass index, blood pressure, total cholesterol, and fasting glucose. All seven lifestyle categories are measured using a scale of 0 to 14, scoring 2 points for ideal, 1 point for intermediate and 0 points for poor quality. The scores are then added and the total score will identify 3 ranks of lifestyle health: 10–14 being optimal, 5–9 as average, and 0–4 being inadequate. The diet components were subjected to content validation by two certified dietitians using the Malaysian Nutrition Guidelines [19]. Local food items which were equivalent in nutritional content were substituted in place of item unfamiliar to the local population (e.g. bran is substituted with brown rice, dark bread, whole grain ready-to-eat cereal).

#### Two Questions with Help Question (TQWHQ)

Questionnaire is self-administered case-finding instrument for depression [20]. This questionnaire has been validated for use at primary care facilities in Malaysia. Respondents who screened positive for depression during recruitment phase are referred for further evaluation and appropriate treatment by Family Medicine Specialists or Psychiatrists, whichever applicable.

### Intervention

The researcher assisted caregivers in the intervention group to install the SRA™ app, and provided individualised training on how to use the app. They had free access to the app including the health education sections during the intervention period and also received the pamphlet. Caregivers could communicate with the care management team about the app through WhatsApp or email.

**Fig. 1:**
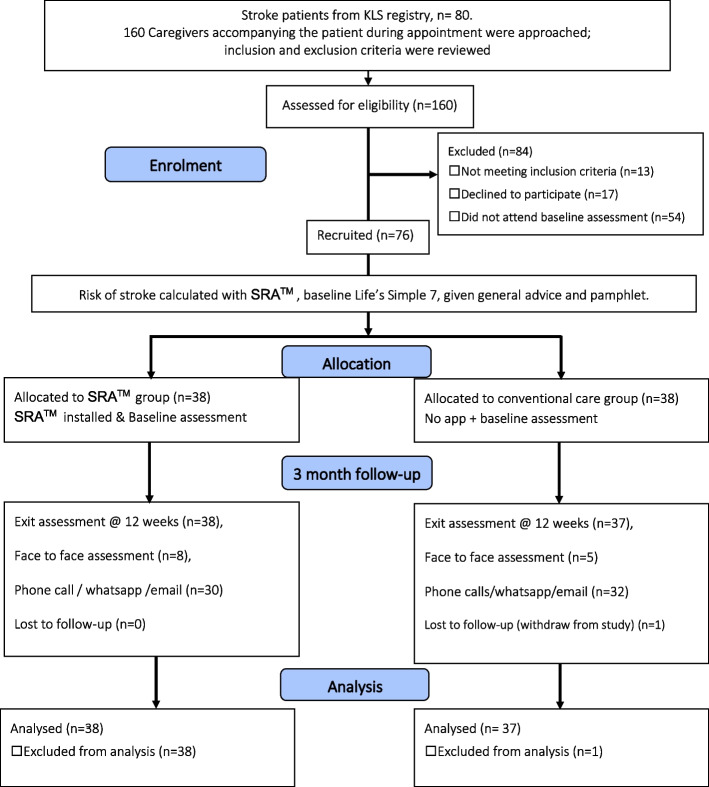
Study flow chart (CONSORT protocol)

The care management team included four members—one medical officer, two staff nurses, and one research assistant. The team received training from the researcher prior to commencement of the trial. The team performed the following functions: provided guidance when required to ensure that the app functioned well, conducted interviews with participants regarding lifestyle according to LS7, tracing blood investigations and informing participants. At baseline, all participants answered the questions in the application, with minimal assistance from researcher. Their absolute and relative risk of getting stroke for the next 5- and 10-years’ time was automatically estimated and standard medical care (motivational education) according to the app-based information was given for those at risk. Standard medical care was defined as treatment provided at primary care level according to Malaysian Clinical Practice Guidelines from Academy of Medicine Malaysia and Ministry of Health (MOH), Malaysia. Standard medical care was given at primary care clinic by registered doctors to all study participants. In addition, participants assigned to the app were able to calculate their own stroke risk and had access to self-help advice embedded in the app and how to act on high-risk domains. The main benefit for interventional group was the ability to learn more about their individual stroke risk with additional opportunity to save their stroke risk profile and monitor changes in their stroke risk over time. Reminders on managing their stroke risk such as exercise and adhering to medication was accessible via the app. Participants in the control group were expected to act on reducing their known risk of getting stroke according to the standard medical care only. Both control and interventional groups was assessed again after 12 weeks by filling up the questionnaire either by email or phone call. During exit assessment only BMI, BP, lifestyle components such as self-reported physical activities were assessed. Due to financial and time constraints, repeat blood investigations (i.e., total cholesterol and fasting blood sugar levels) were not performed (Fig. 1). The review at 12 weeks was timed to coincide with the stroke patients’ follow up schedule at KLS.

The trial was prospectively registered in the Australia-New Zealand Clinical Trial Registry (ACTRN12618002050235, 21/12/2018).

### Primary and secondary outcome measures

Primary objective was to evaluate the impact of Stroke Riskometer Application (SRA™) on lifestyle changes among stroke caregivers using Life Simple 7 scores. Secondary outcomes were to assess caregivers’ stroke risk and relative risk of stroke at 5 and 10 years in the intervention group and Conventional Care group, using SRA™ and to determine lifestyle changes of caregivers within 3 months after knowledge of personal stroke risk, using the LS7 scores. Basically, calculation of absolute and relative risk reduction of stroke were performed in both intervention and conventional group.

### Statistical analysis

Intention to treat principles were used, with last observation carried forward employed for participants who did not turn up for exit assessment. Demographic and risk factor profiles for participants under SRA™ and conventional care were analysed using SPSS version 25 software [21]. All effect estimates were adjusted for prognostic risk factors at baseline, and 95% confidence intervals (CI) were provided. The level of statistical significance was set at *p*-value of < 0.05. Wilcoxon Sign rank test was used to compare pre and post intervention scores of all participants and Mann Whitney test to compare changes between both groups. Multilinear regression analysis was conducted to identify factors which influenced changes in stroke risk, with significance level set at *p* < 0.05.

## Results

The socio-demographic and clinical i.e. risk factor(s) profile of the study participants are detailed in Table 1.

**Table 1:** Demographic characteristics of participants (*N* = 75)

Characteristics	SRA n (%) (*N* = 38)	Control n (%) (*N* = 37)	*p* value
Ages, (Mean±SD) years	46.1 ± 11.3	45.6 ± 12.8	0.87
Gender (n) (%)			
● Female	25 (65.8)	24 (64.9)	0.93
● Male	13 (34.2)	13 (35.1)	
Ethnicity			
● Malay	25 (65.8)	27 (73.0)	0.50
• Non-Malay	13 (34.2)	10 (27.0)	
Education level			
● Primary/secondary	27 (71.0)	24 (64.9)	0.57
• Tertiary	11 (28.9)	13 (35.1)	
Class of occupation			
● Engaged in executive, administrative or clerical duties	18 (47.4)	13 (35.1)	0.05
● Engaged in skilled or semi-skilled work and not exposed to hazardous conditions	8 (21.0)	14 (37.8)	
● Engaged in occupations requiring manual labour or heavy machinery or exposure to certain hazardous conditions	5 (13.2)	9 (24.3)	
• Homemaker	7 (18.4)	1 (2.7)	
Income per month (RM)			
• < 3000	18 (47.4)	21 (56.8)	0.42
• ≥ 3000	20 (52.6)	16 (43.2)	
Residing with stroke patient			
• No	10 (26.3)	11 (29.7)	0.74
• Yes	28 (73.7)	26 (70.3)	
Marital status			
● Married	26 (68.4)	23 (62.2)	0.54
● Single (includes divorced, widowed)	12 (31.6)	14(37.8)	
Relationship to stroke patient			
● First degree (parents, children, siblings) & spouse	31(81.6)	26 (70.3)	0.49
● Second degree (uncle, aunt)	2 (5.3)	4 (10.8)	
● Spouse	5 (13.2)	7 (18.9)	
Hours spent/day providing care for stroke patient			
• < 6 h/day	14 (36.8)	13 (35.1)	0.89
• 6–12 h/day	12 (31.6)	12 (32.4)	
• > 12 h/ day	12 (31.6)	12 (32.4)	
Family history of stroke or heart attack before age 65 years old (%)			
• Yes	33 (86.8)	31 (83.8)	0.71
• No	5 (13.2)	6 (16.2)	
Current active smoker (%)			
• Yes	7 (18.4)	7(18.9)	0.96
• No	31 (81.6)	30 (81.1)	
Baseline BMI (Mean ± SD)	27.1 (6.6)	29.0 (7.0)	0.22
Baseline systolic BP, mmHg (Mean ± SD)	136.9 (14.7)	130.8 (14.2)	0.07
Baseline total cholesterol, mmol/L (Mean ± SD)	5.5 (1.1)	5.6 (1.2)	0.73
Baseline fasting blood sugar, (Mean ± SD)	5.5 (1.2)	5.0 (1.3)	0.09
Performed at least 150 min of physical activity per week (%)			
• Yes	3 (7.9)	2 (5.4)	0.67
• No	35 (92.1)	35 (94.6)	
Eat at least 6 servings of fruits ± vegetables a day (%)			
• Yes	5 (13.2)	2 (5.4)	0.25
• No	33 (86.8)	35 (94.6)	
On regular medication (%)			
• Yes	23 (60.5)	17 (45.9)	0.21
• No	15 (39.5)	20 (54.1)	
Under proper follow up for medical condition			
• Yes	20 (52.6)	24 (64.9)	0.28
• No	18 (47.4)	13 (35.1)	

*p* value: Chi square

### Changes in stroke risk and LS7 scores between groups

Stroke risk: Changes between both groups were not significant (refer Table 2), although within groups, there was a 24.5% reduction in stroke risk at 5 years in the SRA™ group, compared to 16.7% reduction in the conventional care group (Refer table 3). However, these were statistically not significant when we compared between the two groups (Refer table 3). Similar findings were noted in the improvement of stroke risk in the next 10 years (SRA™ 7.1%, conventional care 6.7%).

**Table 2:** Between group differences (SRA^TM^ and control group) at 3 months

**Variables**	**Median (IQR)**	**Median difference (95% CI)**	*P* value*
Stroke risk 5 years (%)			
SRA™	2.04 (1.21)	0.53 (0.15–1.21)	0.13
Control	2.57 (1.70)		
Stroke risk 10 years (%)			
SRA™	3.53 (2.50)	0.81 (0.53–2.15)	0.23
Control	4.34 (3.28)		
Relative risk 5 years			
SRA™	3.42 (1.77)	0.84 (0.29–1.97)	0.14
Control	4.26 (2.99)		
Relative risk 10 years			
SRA™	2.52 (1.23)	0.58 (0.36–1.52)	0.23
Control	3.1 (2.61)		
Life’s Simple 7 scores			
SRA™	9.29 (1.59)	0.88 (1.68–0.08)	0.03
Control	8.41 (1.87)		

^*^Independent T test

**Table 3:** Changes within SRA™ and conventional care group after 3 months

**Variables**	**Median (IQR)**		*p* value*
Stroke risk 5 years (%)			
SRA™	Baseline	2.61 (2.08)	< 0.001
	Exit	1.97 (1.95)	
Control	Baseline	2.76 (2.28)	< 0.001
	Exit	2.30 (2.14)	
Stroke risk 10 years (%)			
SRA™	Baseline	3.35 (3.64)	< 0.001
	Exit	3.10 (3.17)	
Control	Baseline	3.44 (4.10)	< 0.001
	Exit	3.21 (3.75)	
Relative risk 5 years			
SRA™	Baseline	4.00 (3.31)	< 0.001
	Exit	3.37 (2.92)	
Control	Baseline	4.00 (3.88)	< 0.001
	Exit	3.40 (3.14)	
Relative risk 10 years			
SRA™	Baseline	3.00 (1.99)	< 0.001
	Exit	2.72 (2.40)	
Control	Baseline	3.16 (2.07)	< 0.001
	Exit	2.58 (2.02)	
Life’s Simple 7 scores			
SRA™	Baseline	6.50 (2.00)	< 0.001
	Exit	10.00 (3.00)	
Control	Baseline	6.00 (2.50)	< 0.001
	Exit	8.00 (2.00)	
Life’s Simple 5 scores			
SRA™	Baseline	4.00 (2.00)	< 0.001
	Exit	7.00 (2.00)	
Control	Baseline	4.00 (2.50)	< 0.001
	Exit	6.0 2.00)	

^*^Wilcoxon signed rank test

Relative risk of stroke: Reduction was noted in both groups at 5- and 10-years (RR in 5 years- SRA™ 16.5%, Control 15.0%; RR in 10 years- SRA™ 9.3%, Control 18.4%).

Lifestyle changes: At exit assessment, the LS7 total scores showed improvement by 53.8% in the app group with median (IQR) from baseline of 6.5 (2.0) to 10.0 (3.0). As for participants in the control group, their LS7 score showed less improvement from 6 (2.5) baseline to 8 (2.0) at exit, which is a 33.3% improvement. Adjustment was made to analyse the LS7 scores to compensate for unrecorded exit fasting blood sugar and total cholesterol levels.

None of the participants repeated formal blood test at exit interview. Only four participants did capillary blood glucose test at outside facilities i.e. pharmacy/ home glucometer. Readings were informed verbally as “normal value”. Despite the adjustment of using only 5 of the 7 variables, (i.e., LS5 scores), the results remained consistent, with an improvement of 75% seen in the app group while conventional care group showed improvement of 50% in total scores.

Based on multilinear regression analysis, factors which were associated with changes in LS7 scores were body mass index (*p* = 0.001), smoker status (*p* < 0.001) and presence of diabetes at the point of entry into the study (*p* = 0.021). Refer Table 4.

**Table 4:** Multilinear regression analysis of factors affecting LS7 scores between conventional care and SRA™ groups after 3 months

Variables	Unstandardised Coefficients		Standardised Coefficients	t	Sig	95% CI	
	**B**	**Std. error**	**Beta**			**Lower**	**Upper**
(Constant)	9.227	3.027		3.049	.003	3.169	15.286
Control vs SRA™	.429	.315	.121	1.363	.178	-.201	1.058
Age in years	-.013	.017	-.089	-.763	.449	-.048	.021
Ethnic	-.367	.376	-.096	-.977	.333	-1.119	.385
Gender	-.401	.404	-.108	-.991	.326	-1.210	.409
Education level	.787	.442	.207	1.781	.080	-.098	1.672
Current marital status	.095	.215	.041	.443	.659	-.335	.526
Monthly income (RM)	.156	.163	.106	.953	.345	-.171	.483
Class of occupation	.048	.168	.027	.285	.777	-.288	.384
Family history of stroke or heart attack	.179	.477	.036	.376	.709	-.776	1.135
**Classification of BMI**	-.606	.135	-.406	-4.483	**.000**	-.876	-.335
** Smoker**	-1.951	.465	-.429	-4.196	**.000**	-2.882	-1.020
Consume 6 servings of fruits/ vegetables daily	.282	.621	.046	.454	.651	-.962	1.526
Physical activity	1.057	.691	.149	1.529	.132	-.326	2.440
Systolic BP (mmHg)	-.017	.012	-.137	-1.437	.156	-.040	.007
**Diabetes**	.635	.269	.237	2.365	**.021**	.098	1.173
Heart disease	.663	.649	.086	1.021	.312	-.637	1.962

## Discussion

Mobile app-based health promotion programs are said to be an ideal far-reaching platform for efficient interventions because such mobile apps provide an easy way to access the target group [9] and are cost-effective compared to clinic-based or phone-based interventions [22]. They also save time for a clinician for explaining the intervention and allows objective monitoring of the caregivers’ cardiovascular health. The increasing proportions of the population being connected via smartphones and embracing the internet of things (IoT) has provided encouragement as well as challenges towards ensuring a captive and responsive audience in delivery of health education to target groups [11, 13].

Our study has shown that behavioural changes towards more optimum lifestyle practices improved after three months’ exposure to SRA™ particularly in the intervention group. We postulate that stroke caregivers are more receptive when using mobile app rather than face to face counselling, including receiving traditional pamphlets or hardcopy versions of healthcare guidance. This could be due to the fact that the mobile app is user friendly and more patient-oriented, and can be accessed repeatedly at any time of need. The latter feature allows the user to revisit issues which may have been forgotten or requiring further reinforcement of knowledge, at their own time. For the conventional group, we conclude that the close monitoring may have motivated changes in lifestyle practices i.e., Hawthorne’s effect.

Participants with better socioeconomic circumstances in the intervention group may have better access to healthier food choices, especially fruits and vegetables, thereby reducing their risk of NCDs [23]. This is supported by a cohort study among Australians which concluded that women from the lower education group had higher risk of getting stroke as compared to their more educated counterparts, who could afford better, usually more costly food choices [24]. We also postulate that this group would have economical advantage to hire salaried caregivers to take care of the stroke patients, enabling free time to perform physical activities and exercise. On the other hand, although reduction of stroke risk in 5- and 10-years’ time observed between SRA™ and conventional care groups were documented, the differences were not statistically significant. The limited study time frame may have resulted in insignificant cumulative changes in stroke risk and overall lifestyle changes. While multivariate analysis techniques were used to explore relationships among variables and gain preliminary insights, we acknowledge that the results should be interpreted with caution due to the small sample size.

A systematic review of efficacy of interventions using mobile health applications to improve diet and physical activity found that multiple component interventions including face to face counselling in addition to an app, is more effective than a standalone app [23].

Although this study used small number of participants, the observed results are encouraging especially in improving lifestyle practices, thus reducing modifiable risk factors for stroke and other NCDs as well. The findings from this trial support justification for future trials to be conducted across the population, using mobile app in altering risk factors. This study is one of the first to link caregivers’ stroke risk to “objectively measure changes in lifestyle behaviours”. We demonstrate the potential of the SRA™ in facilitating patient empowerment and self-management for NCD such as hypertension. The app not only empowers people to know their absolute risk as well as relative risk of having a stroke within the next 5–10 years, it also encourages reduction of risk of stroke by practicing a healthier lifestyle. The app also educates users about stroke warning signs (extended version of the Face-Arm-Speech-Time (F.A.S.T), their individual and overall risk factors and how to control them by using evidence-based and internationally recognised guidelines [10].

Unlike some expenditures associated with screening of the population for implementation of high-risk prevention strategies, an app-based primary prevention strategy may empower and simultaneously influence the individual’s decision to reduce their risk of stroke. By taking advantage of population-wide and high-risk prevention strategies and at the same time addressing their current limitations, these mobile technologies could be merged into the hospital and community patient management structures, thus providing an important link between patients and healthcare providers.

It was observed that the relative risk of caregivers compared to the general population were in general higher, according to the epidemiological database used by SRA™ [25]. The relative risk calculated in the app was validated on the European epidemiological datasets (ARCOS, Russian, and Rotterdam). Given that a significant proportion of Malaysians engage in infrequent physical activity and fall within the overweight or obese BMI range, coupled with less-than-ideal lifestyle habits aggravated by caregiving responsibilities and familial predisposition, it is probable that there is a heightened risk of cardiovascular disease among family members of stroke patients. To gain a clearer understanding of whether the elevated stroke risk is attributed to caregiving strain or other variables, it is essential to evaluate and account for potential confounding factors, such as caregiving strain.

### Strengths and limitations

One of the strengths of this study was the inclusion of an ethnically diverse urban group of stroke caregivers, who are at increased risk of stroke i.e. the immediate family members of stroke patients. The heterogeneity of the sample population may increase generalizability of results to cardiovascular disease prevention in other settings. However, there were limitations where the short duration of follow up for our study (three months) was inadequate to observe any sustainable improvement in the intervention group. Secondly there was a possible chance of contamination between intervention and control group who might be able to download the app upon exposure during baseline assessment. It is possible that they may have accessed other mobile health application that is similar to SRA™ during the study period (i.e.,7 out of 37 in control arm had downloaded the SRA™). Caregivers among the same family members may be more inspired or motivated to reduce their risk of stroke by adopting a healthier lifestyle and managing their risk factors together as a group.

The main processes that may have led to contamination in this study were health professionals delivering both active and comparator treatments and communication between clinicians and participants from the different trial arms. Contamination can be avoided by carrying out a clustered study design, where clusters were defined by geography, holding treatment sessions at different times in different locations, staggering the arrangement of data collection schedules so that participants do not meet in the waiting room [26]. Communication between participants was thought to be most likely in environments in which participants came into close contact. Examples of this included interaction between participants who were family members and patients in the waiting room. The third limitation was recall bias in self-reporting of lifestyle changes. However, the use of standardized questionnaires and uniformly trained research assistants reduced the likelihood of such bias. The fourth limitation could be attributed to the contact the participants had with the study team, who provided some technical support or guidance to use the app. However, the participants did not require much support hence, it is unlikely that the contact may have contributed to the improvement outcome or response desirability bias.

O Meldevdev and colleagues concluded that the SRA™ consistently captures stroke risk across various countries including Asian. Thus, users can depend on its capability to measure their stroke risks, but as it is a novel stroke prevention approach, the SRA™ should continue to evolve and more research is required to further augment precision and validity of this advantageous tool [26].

Future work should also focus on a qualitative study to explore how the participants felt the mobile application helped in initiating or maintaining the lifestyle changes. A cohort-based study measuring sustainability of life style changes and its impact on stroke risk reduction among Malaysians would support further evidence for its usability and acceptability.

## Conclusion

Relative risk of stroke among familial caregivers is higher than in the general population. Using the SRA™ encouraged familial stroke caregivers to make significant lifestyle changes although the impact in reducing risk of stroke is not significant at three months.

## Data Availability

The datasets used and/or analysed during this study are available from the corresponding author upon reasonable request, and with permission of SRA™ owners (AUT University of Auckland).

## Abbreviations

HCTM: Hospital Canselor Tuanku Muhriz
MOH: Ministry of Health
SRA™: Stroke Riskometer Application
